# Fracture-Pattern-Guided Implant Selection in Extracapsular Proximal Femoral Fractures: A Descriptive Single-Centre Study of Dynamic Hip Screw and Proximal Femoral Nail Fixation in Older Adults

**DOI:** 10.7759/cureus.115320

**Published:** 2026-08-27

**Authors:** Ahmed Mohamed, Srikanth Gandavaram, Vaibhav Bansod, Mohammed Hossain

**Affiliations:** 1 Trauma and Orthopaedics, Royal Lancaster Infirmary, Lancaster, GBR

**Keywords:** ao/ota classification, dynamic hip screw, elderly trauma, extracapsular hip fracture, fracture fixation, hip fracture, intertrochanteric fracture, orthopaedic trauma outcomes, proximal femoral nail, subtrochanteric fracture

## Abstract

Introduction: Dynamic hip screw (DHS) and proximal femoral nail (PFN) are both used to fix extracapsular proximal femoral fractures, with implant selection in routine practice typically guided by fracture morphology. This study describes perioperative outcomes and the pattern of implant selection in older adults treated at a single UK trauma centre.

Materials and methods: This single-centre retrospective descriptive study included all eligible patients aged 65 years or older undergoing fixation of extracapsular proximal femoral fractures during the 12-month study period (1 January to 31 December 2024); all eligible procedures during this period were included, and no sample-size calculation was performed. Data included demographics, American Society of Anesthesiologists (ASA) grade, Arbeitsgemeinschaft für Osteosynthesefragen/Orthopaedic Trauma Association (AO/OTA) fracture classification, time to surgery and mobilisation, length of stay, mechanical complications, reoperation, mortality, and discharge destination. As the groups differed in fracture pattern, with numerical imbalances in age and ASA grade, and were not randomised, statistical comparisons are presented descriptively and should be regarded as exploratory only.

Results: Of 101 procedures in 100 patients (mean age 83.2 years), 78 were treated with DHS and 23 with PFN. Implant selection closely tracked fracture pattern: 98.7% of DHS procedures were for AO/OTA 31-A1/A2 fractures vs 82.6% of PFN procedures for 31-A3 fractures (Fisher's exact p < 0.001). Mobilisation within 24 hours occurred in 64% (DHS) and 48% (PFN); the median length of stay was 20 vs 15.5 days (mean 22.4 vs 28.5 days, the latter skewed by a single 137-day PFN outlier); mechanical complications occurred in five DHS and two PFN patients; reoperation in three and two; 30-day mortality was 7.7% vs 13.0%; and one-year mortality was 39.7% vs 17.4%. Outcome estimates were imprecise due to the small PFN group size and substantial baseline differences between treatment groups.

Discussion: Because implant selection tracked fracture stability rather than randomisation, the groups differed fundamentally in case mix and cannot be treated as comparable cohorts. Baseline differences in fracture morphology and stability, and in comorbidity (most patients were ASA III/IV), offer a plausible, though unadjusted and unconfirmed, alternative explanation for observed outcome differences; the exploratory comparisons presented should not be read as evidence of comparative implant performance.

Conclusions: Implant selection was guided by fracture morphology, consistent with NICE guidance. Comparative effectiveness of DHS vs PFN was not assessed in this non-randomised, fracture-pattern-selected cohort.

## Introduction

Hip fractures represent a major public health concern, particularly among older adults, and are associated with substantial morbidity, mortality, and healthcare expenditure [1]. The majority of these injuries occur following low-energy trauma in patients with osteoporosis and frailty [2]. In the United Kingdom, the incidence of hip fractures continues to rise with an ageing population, placing increasing pressure on healthcare systems [2]. National initiatives such as the National Hip Fracture Database (NHFD) and guidelines from the National Institute for Health and Care Excellence (NICE) have been introduced to standardise care, emphasising timely surgery, early mobilisation, and multidisciplinary management to improve patient outcomes [2,3].

Extracapsular proximal femoral fractures, including intertrochanteric and subtrochanteric patterns, are commonly managed surgically with internal fixation to facilitate early mobilisation. The dynamic hip screw (DHS) is an established method for stable fracture patterns. In contrast, the proximal femoral nail (PFN) is increasingly used for unstable or reverse-obliquity configurations due to its biomechanical properties.

Although several studies have compared outcomes between extramedullary and intramedullary fixation, less is known about how implant selection in routine National Health Service (NHS) practice aligns with fracture morphology and contemporary guideline recommendations.

This descriptive study, undertaken within a single-centre NHS trauma service, therefore aims to: (1) describe implant selection for extracapsular proximal femoral fractures according to Arbeitsgemeinschaft für Osteosynthesefragen/Orthopaedic Trauma Association (AO/OTA) fracture morphology; (2) describe perioperative and postoperative outcomes, including complications, mortality, and discharge destination, in this local cohort; and (3) assess whether observed implant selection was consistent with contemporary NICE guidance. It does not provide evidence to support the comparative effectiveness of either implant.

## Materials and methods

This was a single-centre retrospective descriptive study conducted at Royal Lancaster Infirmary, a district general hospital in the United Kingdom. As implant selection was determined by fracture morphology and surgeon judgement rather than randomisation, this study was designed to describe perioperative outcomes and implant selection practice rather than to compare the effectiveness of DHS and PFN fixation. The study evaluated patients admitted with extracapsular proximal femoral fractures who underwent operative fixation during the study period. Patients admitted between 1 January 2024 and 31 December 2024 were included. All eligible procedures meeting the inclusion criteria during this period were included; no sample-size calculation was performed, as this was a descriptive study of all eligible cases rather than a study designed to detect a pre-specified effect size. Data collection and analysis were performed between December 2025 and April 2026.

Patients were initially identified by members of the study team who collectively reviewed all archived daily trauma operating theatre lists for the full study period (1 January to 31 December 2024); this initial screening was not performed independently or cross-checked between reviewers. Identified cases, along with reasons for exclusion, were recorded in a screening log. Identified cases were subsequently verified in a fixed sequence: patients were first cross-referenced against the PACS imaging archive to confirm fracture type and classification, then against operative notes and clinic correspondence to identify postoperative complications, and finally against GP records and hospital electronic patient records to confirm mortality status and any further complications. Patient identifiers were cross-checked across theatre lists to identify duplicate entries; the one genuine duplicate identified, corresponding to a single patient with bilateral fractures treated as two separate clinical episodes, was marked as such in the screening log and retained as two independent procedures for analysis, as described below. Fractures were confirmed through radiographic review and operative documentation. Patients were included if surgery was performed at Royal Lancaster Infirmary and excluded if surgical fixation occurred at another institution.

The unit of analysis in this study is the surgical procedure, rather than the patient. One patient sustained bilateral extracapsular proximal femoral fractures as two separate clinical episodes approximately four months apart, each with its own presentation, American Society of Anesthesiologists (ASA) grade, perioperative course, and mortality follow-up window; both procedures were treated with DHS and were recorded and analysed as independent procedures. One patient was transferred to another institution following surgery; this patient's 30-day and one-year mortality status was nonetheless confirmed through GP records, in keeping with the mortality verification process described below.

Patients were included if they were 65 years of age or older, sustained an extracapsular proximal femoral fracture, underwent operative fixation, had surgery performed at Royal Lancaster Infirmary, and were treated with either DHS or PFN fixation. Patients were excluded if they sustained pathological fractures, presented with polytrauma (defined as an Injury Severity Score (ISS) greater than 15), sustained periprosthetic fractures, had intracapsular femoral neck fractures, or were managed nonoperatively. True subtrochanteric fractures (AO/OTA 32) were included in the analysis and, for classification and reporting, were grouped with the corresponding 31-A reporting categories based on comparable fracture morphology and fixation strategy.

Fractures were classified according to the AO/OTA classification system [4]. Classification was performed retrospectively by the study authors using the hospital PACS imaging archive. Preoperative radiographs were re-reviewed, and computed tomography (CT) scans were assessed when available to assist with fracture classification. The majority of radiographs were reviewed by a single investigator during data collection.

Implant selection was determined through a combination of NICE Clinical Knowledge Summary guidance and surgeon decision-making [3,5]. Decisions were typically made during the daily trauma multidisciplinary team meeting, which involved consultant orthopaedic trauma surgeons. Fracture morphology played a key role in implant selection. AO 31-A1 fractures were generally treated with DHS fixation, AO 31-A2 fractures were treated with DHS or PFN, with DHS commonly used when the lateral wall was intact, and AO 31-A3 fractures were typically treated with PFN fixation. All intramedullary devices used in the study are referred to as PFN.

Operations were performed by consultant orthopaedic trauma surgeons or orthopaedic registrars under consultant supervision in a standard orthopaedic trauma theatre using fluoroscopic guidance. Patients were positioned on a traction fracture table, and closed reduction was initially attempted under fluoroscopy. If satisfactory reduction could not be achieved, open reduction was performed under fluoroscopic guidance. Patients received perioperative intravenous antibiotic prophylaxis and venous thromboembolism prophylaxis in accordance with institutional protocols. Postoperatively, patients were allowed full weight-bearing as tolerated, and physiotherapy-led mobilisation was initiated from postoperative day 1 where clinically appropriate.

Data were collected retrospectively from multiple sources, including electronic patient records, operative notes, PACS imaging, physiotherapy documentation, ward clinical notes, and clinic correspondence. Recorded variables included patient demographics (age and sex), clinical status measured using the ASA physical status classification, and fracture characteristics classified according to the AO/OTA system. Operative variables included fixation method (DHS or PFN) and time from hospital admission to the start of surgery. Postoperative variables included time to mobilisation, length of hospital stay, and discharge destination. Clinical outcomes recorded were postoperative complications, the requirement for reoperation, and mortality at 30 days and one year.

Time to mobilisation was defined as the time of the first physiotherapy or ward clinical note documenting full weight-bearing activity, such as standing at the bedside or mobilising to the toilet. Documentation indicating transfer to a chair without weight bearing was not considered mobilisation. The primary outcomes assessed were postoperative complications, reoperation, and mortality at 30 days and one year. Reoperation was defined as any subsequent operative procedure related to the index fracture fixation, including revision to arthroplasty (e.g., for implant failure, non-union, or peri-implant fracture) or re-fixation of a peri-implant fracture; it did not include procedures unrelated to the index implant or fixation. Complications, reoperation, and mortality were assessed over a fixed 12-month follow-up period from the date of surgery for all patients. Secondary outcomes included time to surgery, time to mobilisation, length of hospital stay, and discharge destination. Mortality outcomes were verified using hospital and GP electronic records.

Descriptive statistical analysis was performed for all variables. Continuous variables were reported as means with standard deviations (SD), medians, and ranges or interquartile ranges (IQR) where appropriate, while categorical variables were presented as frequencies and percentages. Exploratory statistical comparisons between the DHS and PFN groups were subsequently performed to characterise the dataset descriptively. As implant allocation was determined by fracture pattern rather than randomisation, and the two groups differed markedly in age, ASA grade, and fracture morphology and stability at baseline, these comparisons were not adjusted for confounding and are not intended to test for, or imply, comparative implant effectiveness; p-values and effect estimates should be interpreted as descriptive and exploratory only. Continuous variables (age and length of stay) were assessed for normality using the Shapiro-Wilk test; as both were non-normally distributed, the Mann-Whitney U test was used for between-group comparison. Categorical variables (sex, ASA grade, AO/OTA fracture pattern, postoperative complications, reoperation, 30-day mortality, and one-year mortality) were compared using Fisher's exact test where any expected cell count was below five, or the chi-square test otherwise; in practice, at least one expected cell count was below five for every categorical comparison in this study, given the small size of the PFN group. Fisher's exact test was therefore used throughout. Time to surgery was dichotomised a priori as ≤36 hours versus >36 hours from admission, reflecting the NICE-recommended standard of surgery on the day of, or day after, admission and the corresponding NHFD benchmark, and compared between groups using Fisher's exact test; the full four-category breakdown of time to surgery is reported descriptively in the Results. As one patient contributed two procedures that cannot be considered fully statistically independent, a sensitivity analysis was performed by repeating all comparisons with the second procedure from this patient excluded (n = 100); the results are reported in the Discussion. Discharge destination was dichotomised a priori as discharged home versus not discharged home (nursing home, rehabilitation, transfer to another hospital, or in-hospital death) for statistical comparison, and the groups were compared using Fisher's exact test; the full breakdown of discharge destinations is reported descriptively in the Results. Time to mobilisation was dichotomised a priori as mobilisation within 24 hours versus 24 hours or more, a threshold consistent with commonly used definitions of early mobilisation in the hip fracture literature, and the groups were compared using Fisher's exact test. A p-value of less than 0.05 was considered statistically significant throughout. Statistical analysis was performed using Excel (Microsoft Corp., Redmond, WA, USA) and SPSS Statistics (IBM Corp. IBM SPSS Statistics for Windows. Armonk, NY).

This study was registered and conducted as a clinical audit (registration number: TrOrtho/CA/2025-26/18) using retrospective, anonymised patient data and, in line with the UK Health Research Authority's decision-making guidance, did not require formal approval from a research ethics committee. Institutional clinical audit and governance procedures were followed throughout; individual patient consent was not required given the anonymised, retrospective nature of the data, and patient confidentiality was maintained at all stages.

## Results

During the study period, 101 fixation procedures for extracapsular proximal femoral fractures were performed in 100 patients. All outcomes reported below are analysed at the procedure level rather than the patient level. One patient sustained bilateral fractures as two separate clinical episodes approximately four months apart, each with its own ASA grade, perioperative course, and mortality follow-up period, and both procedures (both DHS) are included as independent observations. Of these, 78 were DHS fixations, and 23 were PFN fixations. All patients were aged 65 years or older, with an overall mean age of 83.2 years (SD 9.0; range 65-100 years). Of the 101 procedures, 77 involved female patients and 24 involved male patients (Table 1).

**Table 1:** Baseline demographic and clinical characteristics of patients undergoing DHS or PFN fixation *One patient sustained bilateral fractures at different time points, accounting for 101 procedures in 100 patients. † Sex is reported by procedure (n = 101); the patient with bilateral fractures (female, DHS) is counted twice. § Length-of-stay data were unavailable for one PFN patient who died during the index admission; figures are based on the remaining 22 patients and include one patient with a prolonged stay of 137 days. ASA: American Society of Anesthesiologists, DHS: dynamic hip screw, IQR: interquartile range, PFN: proximal femoral nail, SD: standard deviation

Characteristic	Overall	DHS	PFN
Procedures, n	101	78	23
Patients, n	100*	78	23
Mean age, years (SD)	83.2 (9.0)	84.1 (8.7)	80.2 (9.7)
Median age, years	-	86	84
Age range, years	65-100	65-100	65-95
Female, n (%)†	77 (76%)	62 (79.5%)	15 (65.2%)
Male, n (%)†	24 (24%)	16 (20.5%)	8 (34.8%)
ASA I, n	-	1	2
ASA II, n	-	8	3
ASA III, n	-	54	14
ASA IV, n	-	15	4
Mean length of stay, days (SD)	-	22.4 (14.2)	28.5 (33.0)§
Median length of stay, days (IQR)	-	20 (13-30)	15.5 (8-35)§
Length of stay range, days	-	1-68	3-137§

In the DHS group (n = 78), there were 62 female and 16 male patients. Mean age was 84.1 years (SD 8.7; median 86 years; range 65-100 years). The majority of patients were classified as ASA III or IV: ASA I, one patient; ASA II, eight patients; ASA III, 54 patients; and ASA IV, 15 patients. Length of stay data were available for all 78 patients: mean 22.4 days (SD 14.2), median 20 days (IQR 13-30 days), range 1-68 days.

In the PFN group (n = 23), there were 15 female and eight male patients. Mean age was 80.2 years (SD 9.7; median 84 years; range 65-95 years). ASA distribution was as follows: ASA I, two patients; ASA II, three patients; ASA III, 14 patients; and ASA IV, four patients (Figure 1). Length-of-stay data were available for 22 of 23 patients; one patient who died during the index admission had no recorded length of stay. Among the 22 patients with data, the mean length of stay was 28.5 days (SD 33.0), the median 15.5 days (IQR 8-35 days), and the range 3-137 days.

**Figure 1:**
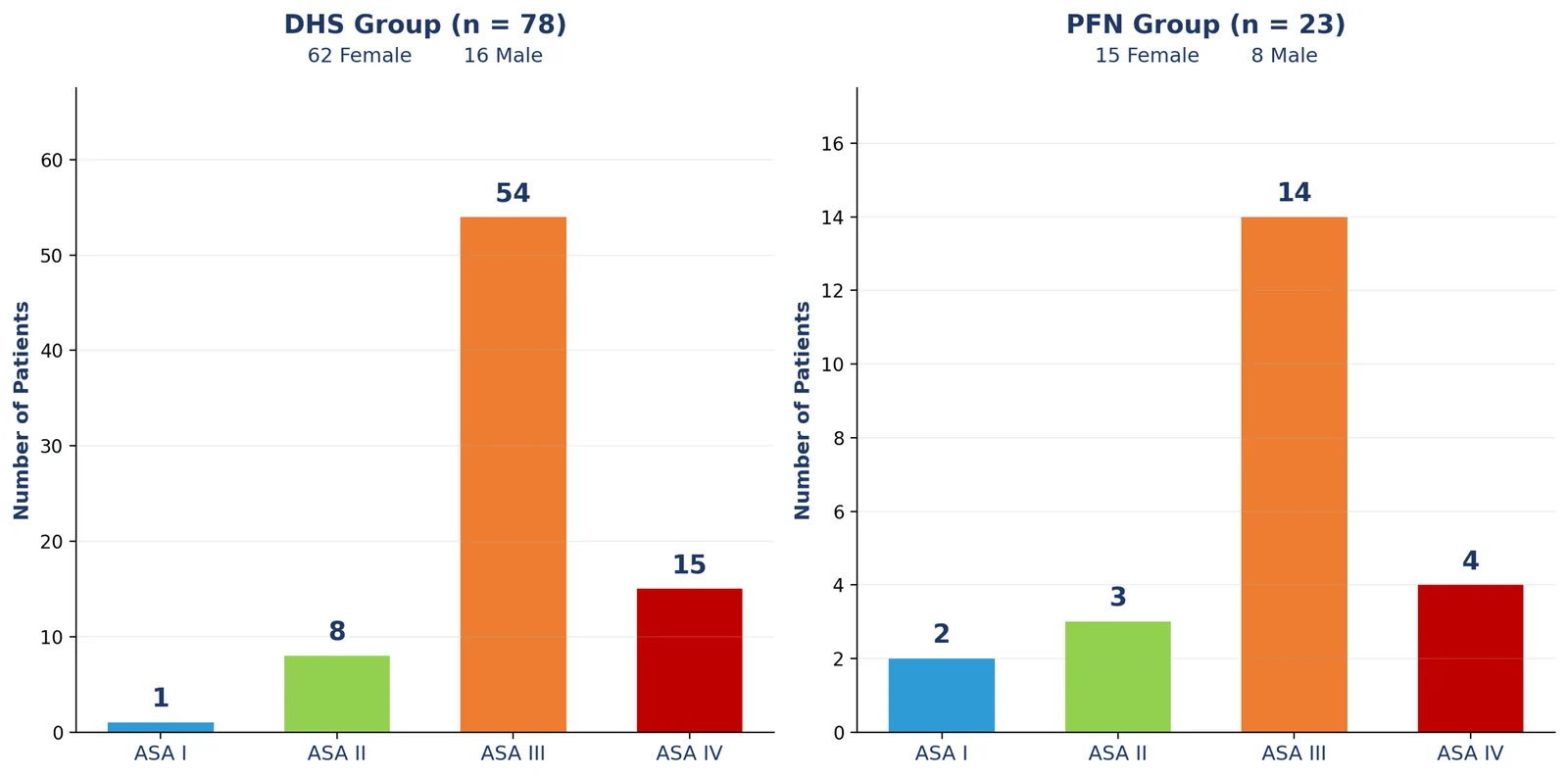
ASA physical status grade distribution by treatment group ASA: American Society of Anesthesiologists, DHS: dynamic hip screw, PFN: proximal femoral nail

Time to surgery, assessed against the NICE-recommended standard of surgery on the day of, or day after, admission (operationalised as ≤36 hours, consistent with the NHFD benchmark), was achieved in 53 of 78 DHS patients (67.9%) and 18 of 23 PFN patients (78.3%). The finer time-to-surgery categories are shown for descriptive completeness: in the DHS group, four patients (5.1%) underwent surgery within six hours of admission, 34 (43.6%) between six and 24 hours, 15 (19.2%) between 24 and 36 hours, and 25 (32.1%) after more than 36 hours; in the PFN group, no patients were operated on within six hours, 12 (52.2%) were treated between six and 24 hours, six (26.1%) between 24 and 36 hours, and five (21.7%) after more than 36 hours.

Time to mobilisation was recorded for all patients. In the DHS group, 50 patients (64%) mobilised within 24 hours of surgery, 21 (27%) between 24 and 48 hours, and four (5.1%) after more than 48 hours; three patients (3.8%) were unable to bear weight and were managed with hoist-only mobilisation. In the PFN group, 11 patients (48%) mobilised within 24 hours, seven (30.4%) between 24 and 48 hours, and four (17.4%) after more than 48 hours; one patient (4.3%) required hoist-only mobilisation.

The fracture pattern was classified according to the AO/OTA system. In the DHS group, 37 fractures (47.4%) were classified as 31-A1, 40 (51.3%) as 31-A2, and one (1.3%) as 31-A3. In the PFN group, one fracture (4.3%) was 31-A1, three (13.0%) were 31-A2, and 19 (82.6%) were 31-A3 (Table 2), reflecting the fracture-pattern-based approach to implant selection in this service.

**Table 2:** Fracture classification according to the AO/OTA system AO/OTA: Arbeitsgemeinschaft für Osteosynthesefragen/Orthopaedic Trauma Association, DHS: dynamic hip screw, PFN: proximal femoral nail

AO/OTA fracture type	DHS, n (%)	PFN, n (%)	Total
31-A1 (stable)	37 (47.4%)	1 (4.3%)	38
31-A2 (comminuted/unstable)	40 (51.3%)	3 (13.0%)	43
31-A3 (reverse obliquity/transverse)	1 (1.3%)	19 (82.6%)	20
Total	78 (100%)	23 (100%)	101

Postoperative mechanical complications were recorded in five DHS patients and two PFN patients. In the DHS group, these comprised peri-implant fracture (n = 2), non-union (n = 2), and implant failure (n = 1); in the PFN group, both patients sustained a peri-implant fracture. Reoperation was required in three DHS patients and two PFN patients. Thirty-day mortality was 7.7% (6/78) in the DHS group and 13.0% (3/23) in the PFN group. One-year mortality was 39.7% (31/78) in the DHS group and 17.4% (4/23) in the PFN group (Figure 2).

**Figure 2:**
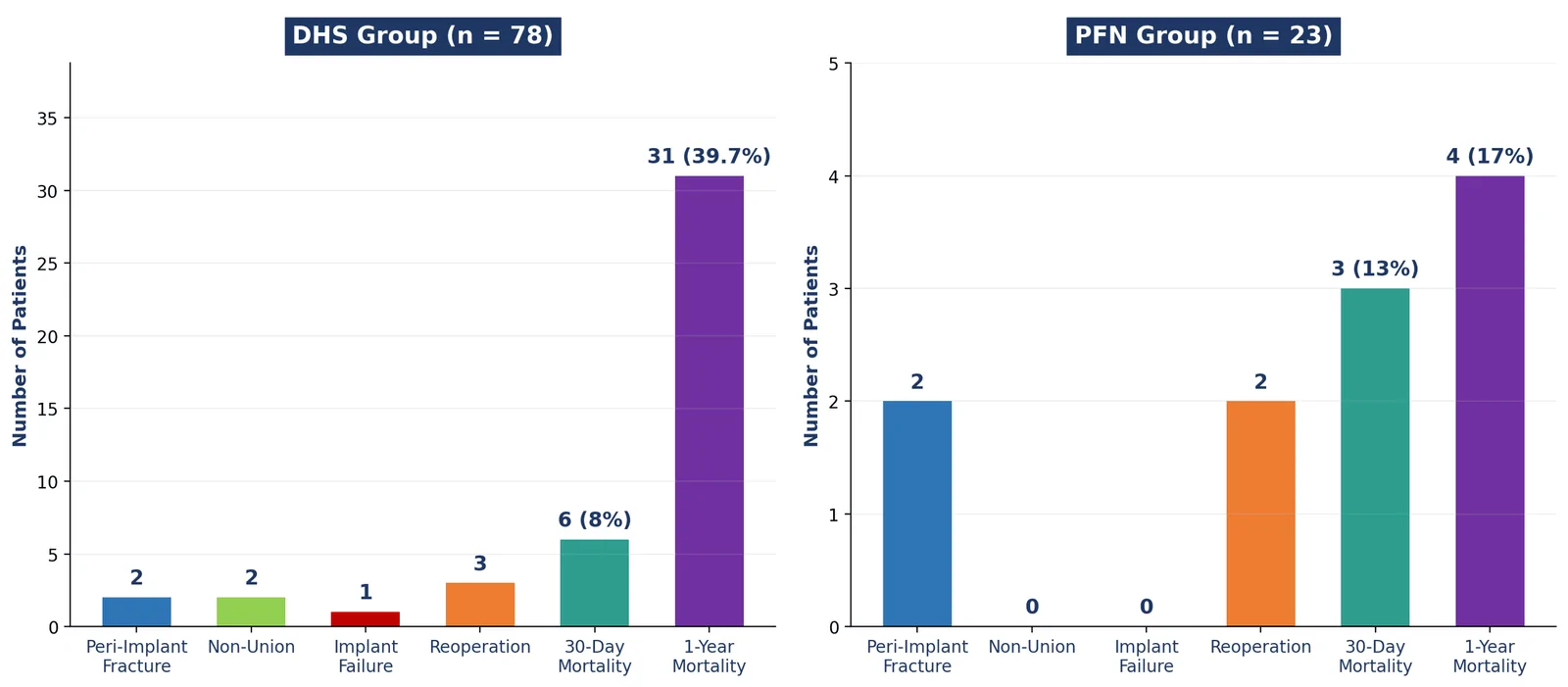
Postoperative mechanical complications and outcomes by treatment group DHS: dynamic hip screw, PFN: proximal femoral nail

Discharge destination was recorded for all patients. In the DHS group, 30 patients (38.5%) were discharged home, 37 (47.4%) to a nursing home, four (5.1%) to rehabilitation, two (2.6%) were transferred to another hospital, and five (6.4%) died during the index admission. In the PFN group, 12 patients (52.2%) were discharged home, seven (30.4%) to a nursing home, two (8.7%) to rehabilitation, and two (8.7%) died during the index admission.

Statistical comparison between groups is summarised in Table 3. These comparisons are exploratory and descriptive in nature: because implant allocation was determined by fracture pattern rather than randomisation, and the two groups differed markedly in age, ASA grade, and fracture morphology and stability at baseline, the reported p-values and effect estimates should not be interpreted as tests of comparative implant effectiveness. Age and length of stay were non-normally distributed (Shapiro-Wilk test) and were compared using the Mann-Whitney U test; categorical variables were compared using Fisher's exact test or chi-square as appropriate. Fisher's exact test was used for all categorical comparisons because at least one expected cell count was below five in each comparison. Fracture pattern was the only variable that differed significantly between groups (Fisher's exact p < 0.001), consistent with fracture-pattern-based implant selection. No other exploratory comparison reached statistical significance, including age, sex, ASA grade, time to surgery, time to mobilisation, length of stay, mechanical complications, reoperation, or 30-day mortality (all p > 0.05). There was no statistically significant difference in the proportion of patients discharged home between groups (p = 0.336); the full breakdown of discharge destinations is reported above and was not formally compared. One-year mortality was numerically higher in the DHS group (39.7% vs 17.4%; Fisher's exact p = 0.079). This unadjusted association cannot be interpreted as an implant effect, because treatment allocation was strongly determined by fracture morphology and the groups differed in baseline characteristics. The estimate was imprecise, and this study was not designed or powered to assess comparative effectiveness.

**Table 3:** Exploratory statistical comparison of baseline characteristics and outcomes between DHS and PFN groups *The test statistic column reports the Mann-Whitney U statistic for continuous variables (age and length of stay) or the OR with 95% CI for Fisher’s exact test comparisons involving 2 × 2 tables. No single test statistic is reported for the AO/OTA fracture pattern comparison because it involves a larger contingency table. ORs represent the odds of the outcome in the DHS group relative to the PFN group (PFN = reference); 95% CIs were calculated using Woolf’s method with Haldane-Anscombe continuity correction when any cell count was zero. An OR >1 indicates higher odds in the DHS group, whereas an OR <1 indicates lower odds. †Statistically significant (p < 0.05). Surgery ≤36 hours reflects the NICE-recommended standard of surgery on the day of or day after admission and the corresponding NHFD benchmark; the full four-category time-to-surgery distribution is reported descriptively in the Results. Comparisons are exploratory and descriptive because implant allocation was based on fracture pattern rather than randomisation, and the groups differed substantially in age, ASA grade, and fracture morphology and stability at baseline. No adjustment for confounding was performed; therefore, results should not be interpreted as evidence of comparative implant effectiveness. The Mann-Whitney U test was used for continuous variables after non-normality was confirmed by Shapiro-Wilk testing. Fisher’s exact test was used for all categorical variables, including AO/OTA fracture patterns, because at least one expected cell count was <5 in each categorical comparison. OR: odds ratio, CI: confidence interval, U: Mann-Whitney U test statistic, AO/OTA: Arbeitsgemeinschaft für Osteosynthesefragen/Orthopaedic Trauma Association, ASA: American Society of Anesthesiologists, DHS: dynamic hip screw, IQR: interquartile range, PFN: proximal femoral nail, NICE: National Institute for Health and Care Excellence

Variable	DHS (n = 78)	PFN (n = 23)	Statistical test	Test statistic*	p-value
Age, years (mean, SD)	84.1 (8.7)	80.2 (9.7)	Mann-Whitney U	U = 1114.5	0.079
Sex (female), n (%)	62 (79.5%)	15 (65.2%)	Fisher's exact	OR = 2.07 (0.75-5.73)	0.172
ASA grade III-IV, n (%)	69 (88.5%)	18 (78.3%)	Fisher's exact	OR = 2.13 (0.64-7.14)	0.300
AO/OTA fracture pattern	-	-	Fisher's exact	-	<0.001†
Surgery <36 h, n (%)	53 (67.9%)	18 (78.3%)	Fisher's exact	OR = 0.59 (0.20-1.77)	0.440
Mobilisation <24 h, n (%)	50 (64.1%)	11 (47.8%)	Fisher's exact	OR = 1.95 (0.76-4.99)	0.225
Length of stay, days (median, IQR)	20 (13-30)	15.5 (8-35)	Mann-Whitney U	U = 913.0	0.650
Any mechanical complication, n (%)	5 (6.4%)	2 (8.7%)	Fisher's exact	OR = 0.72 (0.13-3.98)	0.657
Reoperation, n (%)	3 (3.8%)	2 (8.7%)	Fisher's exact	OR = 0.42 (0.07-2.68)	0.319
30-day mortality, n (%)	6 (7.7%)	3 (13.0%)	Fisher's exact	OR = 0.56 (0.13-2.42)	0.422
1-year mortality, n (%)	31 (39.7%)	4 (17.4%)	Fisher's exact	OR = 3.13 (0.97-10.09)	0.079
Discharge home, n (%)	30 (38.5%)	12 (52.2%)	Fisher's exact	OR = 0.57 (0.22-1.46)	0.336

## Discussion

This single-centre descriptive study demonstrates that implant selection for extracapsular proximal femoral fractures in this NHS trauma service was strongly guided by fracture morphology, with DHS used almost exclusively for AO/OTA 31-A1/A2 patterns and PFN for 31-A3 fractures. Because implant allocation was determined by fracture pattern rather than randomisation, the DHS and PFN groups differ fundamentally in case mix and cannot be treated as comparable cohorts. The discussion below therefore describes the outcomes observed within each group and the factors likely to explain them, rather than testing for comparative implant effectiveness.

The strongest finding in this dataset is the degree of fracture-pattern-based implant selection: DHS was used in 98.7% of 31-A1/A2 procedures, while PFN was used in 82.6% of 31-A3 procedures, a highly significant association (Fisher's exact p < 0.001, used in place of chi-square as the expected cell count for one category fell below five). This is consistent with current NICE guidance [3] and mirrors practice reported elsewhere in the UK literature, including evidence that DHS can still be used selectively in subtrochanteric configurations [6,7]. Implant choice in this service was consultant-led within a daily trauma multidisciplinary team meeting, reflecting routine NHS decision-making rather than a research protocol.

This selection pattern is the single most important methodological consideration in this study. Fracture pattern is both the principal determinant of implant selection and an independent determinant of clinical outcome: unstable fracture patterns are inherently associated with a more difficult perioperative course, regardless of the implant used to fix them. Because the PFN group was composed almost entirely of these inherently higher-risk fracture patterns, the differences reported below in mobilisation, length of stay, complications, mortality, and discharge destination cannot be attributed to implant type; they instead most likely reflect the underlying fracture morphology and stability and case mix that determined implant selection in the first place. This is the central interpretive point of this study and applies to every outcome comparison that follows.

Time to surgery was broadly comparable between groups. NICE guidance recommends surgery on the day of, or the day after, admission [3]; the NHFD Best Practice Tariff operationalises this as surgery within 36 hours of admission, a threshold used for descriptive purposes in this study. Delays beyond 36 hours occurred in both groups: 32.1% of DHS patients and 21.7% of PFN patients underwent surgery after this threshold. No PFN procedures were performed within six hours of admission. This study did not capture the specific reasons for the timing of surgery, so this pattern cannot be attributed to a particular cause. Possible contributing factors include additional imaging review, implant planning, and medical optimisation, which are often required for unstable fracture patterns, though these were not assessed. Delays are unlikely to be attributable to implant type itself.

Early mobilisation within 24 hours was achieved in 64% of DHS patients and 48% of PFN patients, a difference that did not reach statistical significance (p = 0.225). This pattern is consistent with findings from the Scottish Hip Fracture Audit [8] and with subgroup analyses from large UK randomised trials, in which postoperative mobility was more closely linked to fracture stability than to implant type [9,10].

Mean length of stay was numerically shorter in the DHS group (22.4 days, SD 14.2) than in the PFN group (28.5 days, SD 33.0), though this difference was not statistically significant (Mann-Whitney U, p = 0.650). The wide SD in the PFN group is largely driven by one patient with a prolonged stay of 137 days; the median values (20 days vs 15.5 days) indicate a smaller between-group difference than the means suggest. Recent UK registry data have similarly noted that length-of-stay differences between sliding hip screw and intramedullary nail groups are substantially confounded by case mix [8].

Postoperative complications occurred in five DHS patients (peri-implant fracture, n = 2; non-union, n = 2; implant failure, n = 1) and two PFN patients (peri-implant fracture, n = 2). These complication profiles are broadly consistent with established patterns in the literature: extramedullary devices carry a recognised risk of screw cut-out, non-union, and fixation failure, particularly in less stable fracture patterns [11,12], while intramedullary devices carry a recognised risk of peri-implant fracture at the nail tip [13]. Reoperation was required in three DHS patients and two PFN patients, a rate that falls within the 2-10% range reported in comparable UK series [12,14]. Neither the difference in complication rates (p = 0.657) nor reoperation rates (p = 0.319) reached statistical significance; the small absolute number of events in both groups limits the precision of these estimates, and these findings should be regarded as descriptive rather than definitive.

Thirty-day mortality was 7.7% in the DHS group and 13.0% in the PFN group (p = 0.422), both within the range reported nationally by the NHFD [2]. One-year mortality was 39.7% in the DHS group and 17.4% in the PFN group (p = 0.079). The DHS group was also older on average (mean age 84.1 vs 80.2 years) and contained a higher proportion of ASA III/IV patients (88.5% vs 78.3%), both established independent predictors of post-fracture mortality; combined with the underlying fracture-pattern imbalance already noted, this makes it impossible to attribute the observed mortality difference to implant type with the present data. Comparable UK studies have not demonstrated a consistent mortality difference attributable to implant type [8,15]. Outcome estimates for mortality were imprecise given the small PFN group, and this study lacks the precision to determine whether a true difference in one-year mortality exists between groups.

A higher proportion of PFN patients were discharged home (52.2% vs 38.5%), while more DHS patients were discharged to a nursing home (47.4% vs 30.4%). Pre-injury baseline function and residence were not captured in this dataset, and patients selected for PFN based on more complex fracture morphology may nonetheless have been younger and more functionally independent at baseline; these discharge patterns should therefore be interpreted with caution rather than as an implant-related finding.

This study has several strengths worth noting. It captures a cohort of eligible patients treated consecutively within a single NHS trauma service over a defined 12-month period, reflecting real-world consultant-led decision-making rather than a controlled research population. It spans the full spectrum of extracapsular fracture patterns, from stable 31-A1 configurations to unstable 31-A3 and subtrochanteric patterns, and applies descriptive statistical analysis across a comprehensive range of perioperative outcomes to characterise this practice pattern in detail. The outcome domains recorded align closely with those monitored nationally through the NHFD and NICE quality frameworks [2,3], enabling meaningful contextualisation of the local findings against national benchmarks.

The principal limitation of this study is its retrospective, non-randomised design, which is inherent to its purpose as a descriptive study rather than a comparative-effectiveness study. Implant selection was determined by fracture morphology and surgeon judgement rather than randomisation, and the resulting case-mix imbalance means the findings cannot be used to conclude relative implant performance. The PFN group was substantially smaller than the DHS group (23 vs 78 patients), which limited the precision of all outcome comparisons; several clinically meaningful differences, most notably one-year mortality, did not reach statistical significance, but this study lacks the precision to determine whether this reflects true equivalence. Any true difference may have been obscured by the small sample size and baseline case-mix imbalance.

Fracture classification was performed by a single reviewer, without independent second-reviewer assessment, resolution of disagreement, or blinding to implant type, introducing potential observer and classification bias. A sample of radiographs was subsequently reviewed by a senior consultant surgeon, who confirmed agreement with the classifications assigned; however, this did not constitute a formal, randomised inter-rater reliability assessment, and a kappa statistic was not calculated, so observer/classification bias affecting group allocation cannot be excluded.

Similarly, initial case identification from trauma theatre lists was not independently verified by a second reviewer. Exact aggregate numbers at each stage of screening (total records screened, potentially eligible cases, and exclusions by category) were not retained in a form allowing a STROBE-style flow diagram to be reported. In contrast, a screening log recorded individual case-level exclusion reasons; the absence of independently verified aggregate screening totals means that potential selection or ascertainment bias in case identification cannot be ruled out.

True subtrochanteric fractures (AO/OTA 32) were grouped with the corresponding 31-A reporting categories for this analysis. Although this grouping reflects a comparable fixation strategy, it introduces some heterogeneity within each reported fracture-pattern group and may limit the granularity of the fracture-pattern analysis presented. NICE guidance recommends intramedullary nailing for both reverse-obliquity/transverse (31-A3) and subtrochanteric fracture patterns, so this grouping does not misrepresent the recommended fixation strategy; however, NICE and the NHFD monitor subtrochanteric fractures as a distinct quality metric separate from other trochanteric patterns. As true subtrochanteric (AO/OTA 32) fractures were not recorded as a distinct category at the point of original data classification, they cannot be separated from the 31-A3 group retrospectively, and this study cannot report against that specific national quality metric.

The unit of analysis throughout this study is the procedure rather than the patient; one patient contributed two DHS procedures as separate clinical episodes several months apart. Although each episode had its own independent presentation and follow-up period, these two observations cannot be considered fully statistically independent, and this represents an additional limitation of the exploratory statistical comparisons presented. As a sensitivity analysis, all comparisons were repeated, excluding the second episode from this patient (n = 100). Results were materially unchanged across all comparisons except one-year mortality, for which the p-value shifted from 0.079 to a nominally significant 0.049; given how close both values are to the significance threshold and the small numerical change involved, this shift should not be overinterpreted but is reported here for transparency.

This study captured only mechanical complications; medical complications such as delirium, pneumonia, acute kidney injury, venous thromboembolism, and cardiac events were not systematically recorded, despite being important contributors to postoperative morbidity, mortality, time to mobilisation, and discharge destination after hip fracture. Similarly, factors known to influence these outcomes beyond ASA grade alone, including baseline functional status, frailty, bone quality, and overall comorbid disease burden, were not captured. No functional outcome measures, radiographic assessments, or pre-injury baseline data were recorded. These unmeasured factors are likely to be important drivers of the reported outcomes and substantially limit the ability to fully contextualise recovery, mortality, and discharge outcomes.

Given these baseline imbalances, multivariable adjustment or a sensitivity analysis restricted to AO/OTA 31-A2 fractures (the only pattern treated with both implants) could in principle have been attempted; however, with only three PFN patients in this subgroup, such analyses would have been insufficiently powered to yield meaningful estimates. All statistical comparisons in this study are therefore presented as descriptive and exploratory only and are not adjusted for confounding by age, ASA grade, or fracture morphology and stability.

Taken together, these findings describe a pattern of practice in which DHS is used for stable 31-A1 and most 31-A2 fractures, and PFN for unstable 31-A3 and reverse-obliquity configurations. This pattern is consistent with NICE guidance [3], systematic review evidence for A3 fractures specifically [7], and Cochrane-level evidence, which has not demonstrated consistent superiority of cephalomedullary nailing over extramedullary fixation across fracture types as a whole and instead emphasises selection by fracture pattern [16]. This study does not itself provide comparative-effectiveness evidence for DHS versus PFN; rather, it describes a fracture-pattern-guided selection practice that appears consistent with the existing evidence base.

A genuine comparative-effectiveness evaluation of DHS versus PFN would require a prospective, ideally multicentre or randomised, study design with a matched or adjusted case mix, larger PFN cohorts, validated functional outcome measures, radiographic assessment of fixation quality, and pre-injury baseline function. Such work falls outside the scope of the present descriptive study but would be a valuable next step in this area.

## Conclusions

In this single-centre descriptive study, implant selection for extracapsular proximal femoral fractures followed fracture morphology, consistent with current NICE guidance: DHS was used almost exclusively for stable AO/OTA 31-A1/A2 patterns, and PFN predominantly for unstable 31-A3 configurations. Comparative effectiveness of DHS vs PFN was not assessed in this study. The non-randomised, fracture-pattern-selected design, together with the absence of functional and radiographic outcome data, means that outcome differences between groups, including differences in mortality and discharge destination, cannot be attributed to implant choice and most likely reflect unmeasured differences in baseline case mix, comorbidity, and pre-injury function between groups.

Future work in this area would benefit from prospective, ideally multicentre, study designs with a larger cohort of patients treated with intramedullary fixation, validated functional outcome measures, radiographic assessment of fixation quality, and pre-injury baseline function. Such designs, potentially incorporating multivariable adjustment or a sensitivity analysis restricted to fracture patterns treated with both implants, would be needed to assess comparative implant effectiveness, which lies outside the scope of the present study.
